# Incidental Finding of Unilateral Tensor Fascia Lata Agenesis in a Marathon Runner: An Unreported Phenomenon

**DOI:** 10.3390/diagnostics15182396

**Published:** 2025-09-20

**Authors:** Tommaso Bellini, Claudio Bruno, Giacomo Brisca

**Affiliations:** 1 Pediatric Emergency Room and Emergency Medicine Unit, Emergency Department, IRCCS Istituto Giannina Gaslini, 16147 Genoa, Italy; tommasobellini@gaslini.org; 2 Centre of Translational and Experimental Myology, IRCCS Istituto Giannina Gaslini, 16147 Genoa, Italy; claudiobruno@gaslini.org; 3 Department of Neurosciences, Rehabilitation, Ophthalmology, Genetics and Maternal and Child Health (DINOGMI), University of Genoa, 16147 Genoa, Italy; 4 Neonatal and Pediatric Intensive Care Intermediate Care Unit, Emergency Department, IRCCS Istituto Giannina Gaslini, 16147 Genoa, Italy

**Keywords:** incidental finding, marathon runner, muscle agenesis, muscle MRI, tensor fascia lata

## Abstract

Congenital agenesis of the tensor fascia lata (TFL) muscle is an extremely rare anomaly, with very few reports in the literature and unclear clinical significance. We report the incidental finding of unilateral TFL agenesis in a 25-year-old male physician who had been enrolled as a healthy control in a muscle MRI study on genetic myopathies. Imaging demonstrated a complete absence of the right TFL with mild compensatory hypertrophy of the ipsilateral rectus femoris, while the contralateral side and all other muscles appeared normal. The subject had no history of neuromuscular disease, exhibited only a subtle waddling gait, and had previously completed the New York Marathon in 4 h and 16 min without symptoms. Laboratory tests, including creatine kinase, were within normal limits. Thirteen years later, he remains in good health, continues regular sports activities, and has not developed pain or functional impairment. This case emphasizes that TFL agenesis may remain clinically silent and compatible with high levels of physical activity. Nevertheless, awareness of such anomalies is important, as compensatory mechanisms might predispose to long-term biomechanical imbalance, and recognition on imaging can prevent misinterpretation or unnecessary investigations

**Figure 1:**
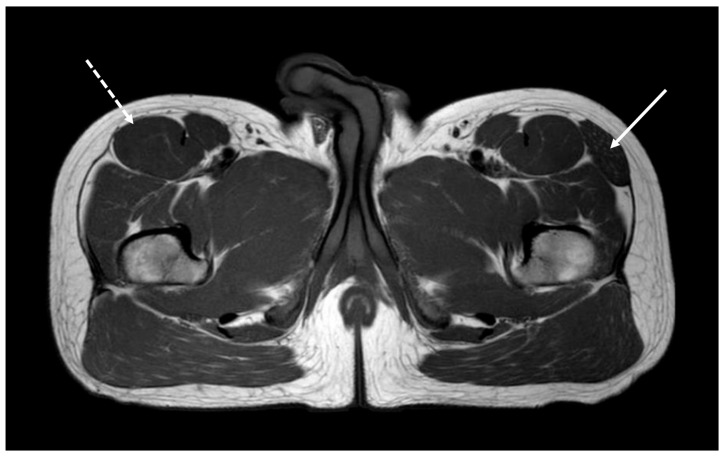
A 25-year-old physician born from non-consanguineous parents was included as a healthy control in a muscle-MRI study conducted by the third author (G.B.) on genetic myopathies as a part of his pediatric residency thesis. T1-weighted axial sections performed at the lower girdle level surprisingly showed the agenesis of the right TFL with mild compensatory hypertrophy of the ipsilateral rectus femoris (dashed arrow) and unaffected surrounding tissues. A normal appearance of the TFL was noted on the left side (arrow). Muscle MRI was bilaterally normal at thigh and leg levels. The tensor fasciae lata (TFL) is a muscle of the proximal anterolateral thigh that lies between the superficial and deep fibers of the iliotibial (IT) band [1]. There is high variability in muscle belly length, although, in most patients, the TFL muscle belly ends before the greater trochanter of the femur [1,2]. The exact role of TFL is not entirely clear. However, it cooperates with the gluteus maximus, gluteus medius, and gluteus minimus in various hip movements, including flexion, abduction, and internal rotation [1]. Furthermore, TFL acts via the IT band’s attachment to the tibia to assist with knee flexion and lateral rotation. The TFL can be considered clinically important for assisting pelvic stability while standing and walking [1]. Although rarely reported, conditions associated with TFL impairment may present as pain and dysfunction in the hip, pelvis, and spine region [3]. These conditions include benign and malignant lesions, such as lipoma, liposarcoma, intramuscular lymphoma and metastases, arteriovenous malformations, muscular atrophy, and adipose pseudohypertrophy [3]. Congenital agenesis of the TFL muscle is an extremely rare anomaly in which the muscle fails to develop during embryogenesis due to unknown reasons. Very little is known about the impact of TFL absence on human life [4,5].

**Figure 2:**
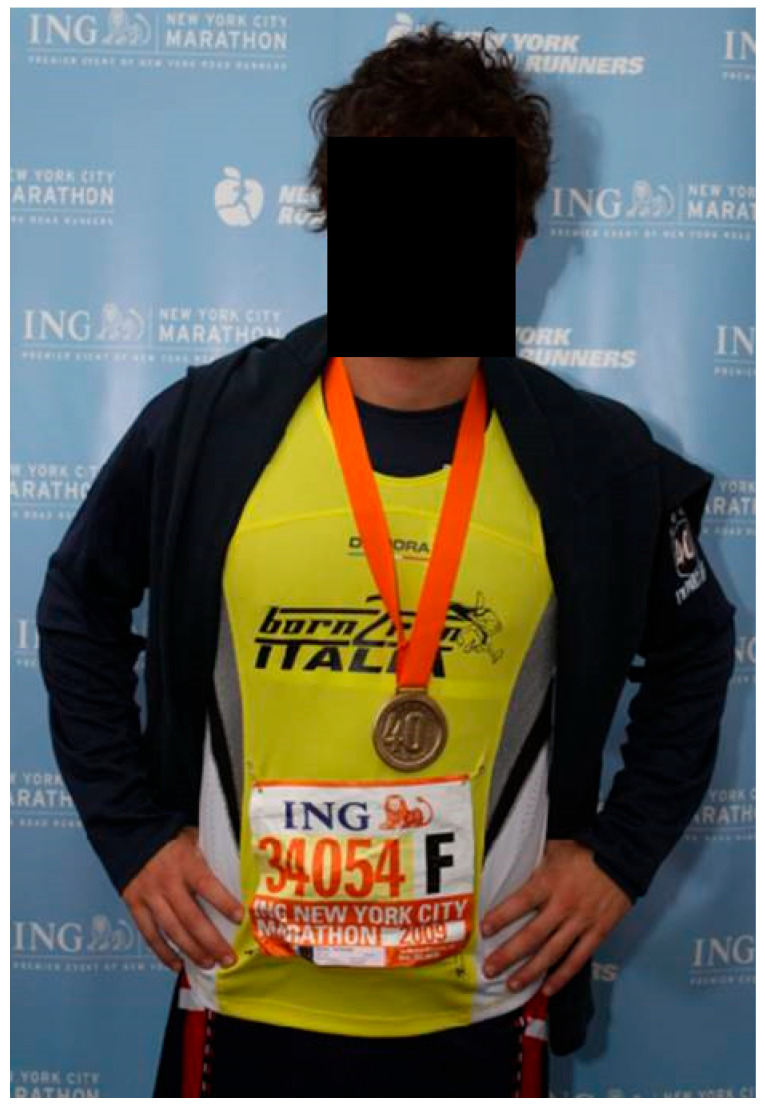
Two years earlier, T.B. had participated in the New York Marathon, unaware of his defect, completing the course in 4 h and 16 min (Figure 2). He had always led an unrestricted sporting life with no particular symptoms due to exercise. However, neurological evaluation pointed out a very slight waddling gait with normal strength tests. He had no signs of neuromuscular or other diseases. Creatine-kinase levels were in the normal range. Electromyography was not performed. Exploring his medical history, his parents reported that he likely did not crawl as an infant, a typical developmental milestone that, in light of our findings, could be associated with the normal development of the TFL muscle. Thirteen years after the marathon, T.B. is in good general health, has an unchanged clinical neurological examination, regularly plays sports, and continues not to complain of painful symptoms after exercise. Agenesis of TFL is rarely reported in the literature [4,5] and when present, it is often discovered incidentally during diagnostic tests for other conditions. For this reason, the real incidence is reasonably underestimated and not known. As shown in our report, individuals with TFL agenesis can lead normal lives and even engage in strenuous activities without any apparent difficulty, as the body may compensate by utilizing other muscles that perform similar functions. However, long-term monitoring is needed, as, over time, this condition could lead to increased fatigue, muscle pain, or difficulty with movements involving the hip. In some cases, therapeutic interventions focused on symptom management and rehabilitation may be necessary to improve lower limb mobility and function.

In this context, our case also highlights how variability in human muscle development can remain clinically silent, even in rare congenital anomalies such as TFL agenesis. This observation underlines the importance of considering musculoskeletal variations during MRI studies, since subtle or uncommon defects may otherwise be overlooked both in clinical practice and in research.

## Data Availability

The data are available upon reasonable request.

## Abbreviations

The following abbreviations are used in this manuscript:

TFL	Tensor Fascia Lata
IT	Ilio-Tibial

## References

[1] Trammell A.P., Nahian A., Pilson H. (2023). Anatomy, Bony Pelvis and Lower Limb: Tensor Fasciae Latae Muscle. StatPearls.

[2] Cho K.H., Jin Z.W., Abe H., Wilting J., Murakami G., Rodríguez-Vázquez J.F. (2018). Tensor fasciae latae muscle in human embryos and foetuses with special reference to its contribution to the development of the iliotibial tract. Folia Morphol..

[3] Iyengar K.P., Azzopardi C., Kiernan G., Botchu R. (2022). Isolated pathologies of Tensor Fasciae Latae: Retrospective cohort analysis from a tertiary referral centre. J. Clin. Orthop. Trauma.

[4] Meberg A., Skogen P. (1987). Three different manifestations of congenital muscular aplasia in a family. Acta Paediatr. Scand..

[5] Randelli F., Papavasiliou A., Mazzoleni M.G., Fioruzzi A., Basile G., Ganz R. (2021). Femoral head necrosis and progressive osteoarthritis of a healed intracapital osteotomy in a severe sequelae of Legg-Calvé-Perthes disease with aplasia of tensor fasciae latae. J. Hip Preserv. Surg..

